# Effect of Particle Heterogeneity in Catalytic Copper-Containing Single-Chain Polymeric Nanoparticles Revealed by Single-Particle Kinetics

**DOI:** 10.3390/molecules29081850

**Published:** 2024-04-18

**Authors:** Anjana Sathyan, Emmanouil Archontakis, A. J. H. Spiering, Lorenzo Albertazzi, Anja R. A. Palmans

**Affiliations:** 1 Department of Chemical Engineering and Chemistry, Institute for Complex Molecular Systems, Eindhoven University of Technology, P.O. Box 513, 5600 MB Eindhoven, The Netherlands; a.sathyan@tue.nl (A.S.); a.j.h.spiering@tue.nl (A.J.H.S.); 2 Department of Biomedical Engineering, Institute for Complex Molecular Systems, Eindhoven University of Technology, P.O. Box 513, 5600 MB Eindhoven, The Netherlands; arxontakis6@gmail.com (E.A.); l.albertazzi@tue.nl (L.A.)

**Keywords:** single-chain polymeric nanoparticles, catalysis, single-particle catalysis, depropargylation, single-molecule fluorescence microscopy

## Abstract

Single-chain polymeric nanoparticles (SCPNs) have been extensively explored as a synthetic alternative to enzymes for catalytic applications. However, the inherent structural heterogeneity of SCPNs, arising from the dispersity of the polymer backbone and stochastic incorporation of different monomers as well as catalytic moieties, is expected to lead to variations in catalytic activity between individual particles. To understand the effect of structural heterogeneities on the catalytic performance of SCPNs, techniques are required that permit researchers to directly monitor SCPN activity at the single-polymer level. In this study, we introduce the use of single-molecule fluorescence microscopy to study the kinetics of Cu(I)-containing SCPNs towards depropargylation reactions. We developed Cu(I)-containing SCPNs that exhibit fast kinetics towards depropargylation and Cu-catalyzed azide-alkyne click reactions, making them suitable for single-particle kinetic studies. SCPNs were then immobilized on the surface of glass coverslips and the catalytic reactions were monitored at a single-particle level using total internal reflection fluorescence (TIRF) microscopy. Our studies revealed the interparticle turnover dispersity for Cu(I)-catalyzed depropargylations. In the future, our approach can be extended to different polymer designs which can give insights into the intrinsic heterogeneity of SCPN catalysis and can further aid in the rational development of SCPN-based catalysts.

## 1. Introduction

Enzymes are nature’s macromolecular biological catalysts that show remarkable activity, selectivity, and specificity, and they have been fine-tuned through millions of years of natural selection [1]. Their intricate three-dimensional (3D) structures and precise positioning of catalytic residues for specific substrate binding have made them an ideal template for the design of synthetic catalysts. Chemists have long sought to mimic them [2,3,4], and one such approach has focused on the folding of synthetic polymer chains around metal catalysts to form catalytic single-chain polymeric nanoparticles (SCPNs) [5,6,7,8,9]. Despite great advances in the field, there are still only a few reports where catalytic SCPNs have shown enzyme-like behavior such as selective substrate binding, high reaction rates, and high activity in competitive conditions [6,7,10,11]. The catalytic performance of SCPNs lags significantly behind that of enzymes, which has been attributed to their inherent structural heterogeneity that arises from the stochastic incorporation of the different monomers and catalytic moieties and the lack of discreteness in the molecular weights of the copolymers [12]. As a consequence, each individual SCPN can be expected to exhibit unique catalytic activity and selectivity, owing to the non-uniform distribution of catalytic sites in different microenvironments [12]. Quantitative analysis of the catalytic activity of SCPNs at the ensemble level only provides an average value of their catalytic activity, which limits our understanding of the diversity of catalytic potential at the single-polymer level. Therefore, developing a technique to directly monitor the catalytic activity of individual SCPNs is beneficial to gain insights into the effect of structural heterogeneities on their catalytic performance. Such understanding can help to rationally design SCPN-based catalysts in the future [12]. 

Single-molecule fluorescence microscopy (SMFM) is a valuable tool to reveal intricate details of different biological processes, which are hidden in ensemble measurements. Some among the many applications of this technique include the tracking of biomolecules in cells [13], monitoring binding kinetics [14], protein folding and movement [15,16], and single-enzyme kinetics [17]. Enzymes were immobilized on a glass surface to study their kinetic behavior at single-turnover resolution by Xie and co-workers [17]. When non-fluorescent substrates are converted to fluorescent products, fluorescent bursts can be visualized using a sensitive fluorescence microscopy method like total internal reflection fluorescence (TIRF) microscopy. The frequency and intensity of these bursts can be used to determine the catalytic activity of individual enzymes. Each fluorescent burst indicates the formation of the product, which is a turnover event. As a consequence, the frequency of the bursts directly yields the turnover frequency (TOF) of the enzymes [18,19]. Even in enzymes, which are monodisperse and structurally homogenous, minor fluctuations in catalytic activity can be probed by single-molecule catalysis, especially the temporal fluctuations that are impossible to track during ensemble measurements [1]. Single-molecule approaches for single-enzyme studies have also been employed to study nano- and microscale heterogeneous catalysts [20,21,22,23,24]. In the pioneering work of Chen and co-workers, the heterogeneous reactivity of Au nanoparticles at the single-molecule level was studied. Their results revealed activity fluctuations over time due to both catalysis-induced and spontaneous surface restructuring of the catalysts [20].

When it comes to SCPNs, single-molecule approaches will be highly beneficial to probe the intrinsic and temporal heterogeneity in their catalytic activity. This requires the development of catalysts with high turnover frequency (TOF) to obtain good statistics for a reliable interpretation of the data [12]. Despite the challenges in attaining sufficiently high TOF in synthetic systems, it is worthwhile to start taking the crucial first steps so that a deeper understanding of the heterogeneity in synthetic compartmentalized catalysts can be gained. Recently, we succeeded in successfully probing the single-particle polarity of SCPNs using Nile Red-based spectral point accumulation for imaging in nanoscale topography (NR-sPAINT). This study unveiled the heterogeneity in polarity within the individual nanoparticles (intraparticle heterogeneity), between particles of the same polymer batch (interparticle heterogeneity), and between particles arising from different polymer designs [25]. In addition, Liu et al. looked at the single-particle kinetics of catalytic SCPNs using confocal microscopy. Catalytic Cu-SCPNs were successfully immobilized on the surface of glass coverslips and were found to retain their catalytic activity. However, issues such as low TOF of the catalyst and low signal-to-noise ratio in measurements were encountered [12]. 

To overcome the above-mentioned challenges, we here introduce the use of single-molecule fluorescence microscopy to study SCPN kinetics at the single-particle level. Towards this, we develop Cu(I) SCPNs that exhibit fast kinetics towards depropargylations and Cu-catalyzed azide-alkyne click (CuAAC) reactions. We select depropargylations as monomolecular reactions that are of high importance for prodrug activation [26,27,28]. The bimolecular CuAAC reactions are selected because they are studied in similar systems and can serve as a good benchmark [7,8]. We assessed the TOF at the ensemble level for both types of reactions. Finally, Cu(I) SCPNs were immobilized on coverslips and their activation kinetics were studied at the single-particle level. The preliminary results of single-molecule catalysis revealed the heterogeneity in catalytic activity between different SCPNs within the same batch. This work emphasizes the possibility of taking steps towards establishing a correlation between structural heterogeneity and function in catalytic single-chain polymeric nanoparticles.

## 2. Results

### 2.1. Design, Synthesis, and Characterization of Cu(I) SCPNs

**Scheme 1:**
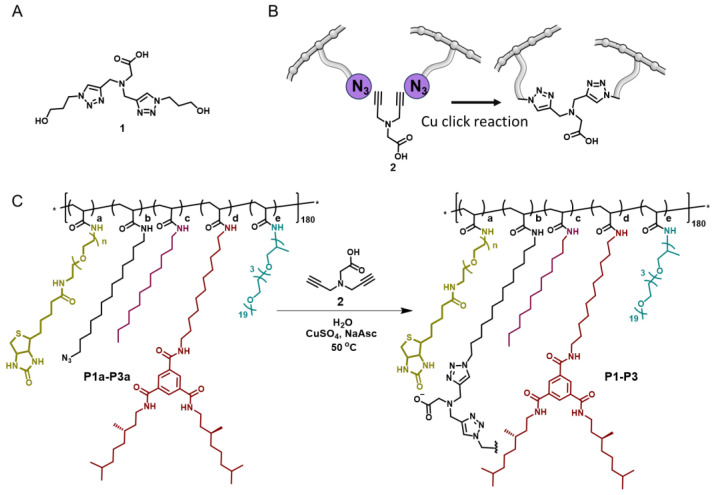
(**A**) Chemical structure of glycine-derived Cu(I)-binding ligand **1**; (**B**) incorporation of ligand to polymer backbone by copper-catalyzed azide-alkyne click reaction; (**C**) chemical structures of amphiphilic polymers **P1a**−**P3a** functionalized with biotin, dodecyl azide, dodecyl, BTA, and Jeffamine@1000 and the subsequent incorporation of diyne **2** to obtain ligand-functionalized amphiphilic polymers **P1**−**P3** by CuAAC reaction.

The first step towards the design of catalytic SCPNs with high TOF is the selection of appropriate ligands for the Cu catalyst that afford high TOF in depropargylation or CuAAC reactions. Glycine derivative ligand **1** (gly) has recently been reported to enhance the reaction rate of CuAAC reactions [29]. We envisaged a similar rate acceleration when this ligand is applied in a depropargylation reaction. To incorporate the gly ligand **1** into the amphiphilic polymer backbone, a covalent strategy was used as previously reported by Zimmerman and co-workers (Scheme 1) and which intramolecularly crosslinks the polymer chain into an SCPN [6]. This strategy allows for the facile incorporation of ligand **6**, leaving the carboxylate anion free to give additional stabilization for the Cu(I) ion. The SCPNs were prepared starting from poly(pentafluorophenyl acrylate) homopolymer (***p*PFPA_180_**, degree of polymerization (DP) = 180, and molar mass dispersity (*Ð*) = 1.19) following protocols previously reported by our group [30]. This polymer was functionalized with amine-based grafts to make polymer precursors **P1a**−**P3a** with the following pendant groups: (i) biotin for immobilizing catalytic SCPN on streptavidin functionalized coverslips for the single-molecule studies, (ii) *n*-dodecyl azide to allow for intramolecular crosslinking with diyne **2** via CuAAC to install the gly **1** ligand into the polymer, (iii) *n*-dodecyl for additional hydrophobicity, (iv) benzene-1,3,5-tricarboxamides (BTAs) for inducing a structured hydrophobic interior via hydrogen-bond formation, and (v) Jeffamine@M-1000 for imparting hydrophilicity. Amphiphilic polymers **P1a**−**P3a** comprised varying degrees of pendant groups as shown in Scheme 1. Table 1 summarizes the details of the polymer compositions. We aimed for an incorporation of around 3% biotin and 20% dodecyl azide in all polymers **P1a**−**P3a**. The amounts of the hydrophobic grafts were varied, where **P1a** only comprises dodecyl azide for hydrophobicity, **P2a** has an additional 5% BTA for a more structured hydrophobic interior [31], and **P3a** has 15% dodecyl instead of BTA (Table 1, Scheme 1). We hereby can assess if the changes in the hydrophobic interior affect the kinetics of the reaction both at the ensemble as well as at the single-particle level. 

Amphiphilic polymers **P1a**−**P3a** were first formulated into SCPNs in water (20 mg/mL) and were then intramolecularly crosslinked using diyne **2** by a CuAAC reaction according to a reported protocol [6]. Since **P1a**−**P3a** collapse/fold in water into small, compartmentalized nanoparticles, the crosslinking will predominantly occur intramolecularly and within one single polymer chain [32]. After the reaction, copper was removed using a Chelex resin and the polymers were dialyzed in water for 5 days to obtain **P1**−**P3**. The incorporation of the ligand was confirmed by IR spectroscopy by the disappearance of the peak at 2096 cm^−1^ corresponding to the azide group (Figure S8). Polymer **P4** without a Cu-binding ligand, which contains only 20% dodecyl and 80% Jeffamine@M-1000, was used as control (Scheme S1).

The size of the polymeric nanoparticles before and after the incorporation of the ligand was monitored using dynamic light scattering experiments (DLS). All polymer precursors **P1a**−**P3a** formed nanoparticles in water with a hydrodynamic radius (*R*_H_) of 4–5 nm. After incorporation of the gly **1** ligand, **P1**−**P3** showed the same or slightly smaller hydrodynamic radii of 3–5 nm, confirming the formation of intramolecularly crosslinked SCPNs (Table 1, Figures S11 and S12). Size exclusion chromatography (SEC) of the polymer precursors **P1a**−**P3a** in DMF indicated that all polymers have a similar molar mass dispersity (*Ð*) of 1.2. After ligand incorporation, SEC was performed in PBS. **P1**−**P3** showed a similar *Ð* to that of their precursors (*Ð* =1.3) (Figure S9). An overview of *R*_H_ and *Ð* of all polymers can be found in Table 1. 

### 2.2. Catalytic Efficiency of Cu(I) SCPNs—Depropargylation and CuAAC Reactions

**Figure 1:**
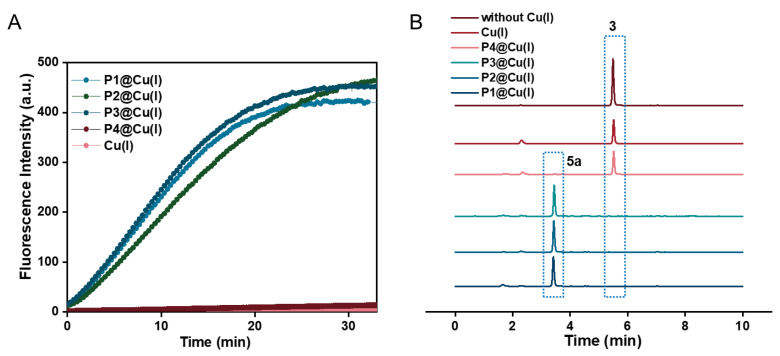
(**A**) Fluorescence kinetic profile of CuAAC reaction between **3** and **4a** to form **5a** using **P1**−**P3@Cu(I)**, **P4@Cu(I),** and Cu(I), in H_2_O, [Cu(I)] = 10 µM, [3] = 100 µM, [**4a**] = 400 µM, [NaAsc] = 2 mM, [**P**] = ~600 nM, at 37 °C, λ_ex_ = 340 nm, and λ_em_ = 470 nm; (**B**) HPLC-UV chromatogram after 35 min, showing complete disappearance of **3** and formation of **5a** (which is more hydrophilic than azide **3**) in the case **of P1**−**P3@Cu(I)**, monitored at λ_ex_ = 340 nm.

**Scheme 2:**
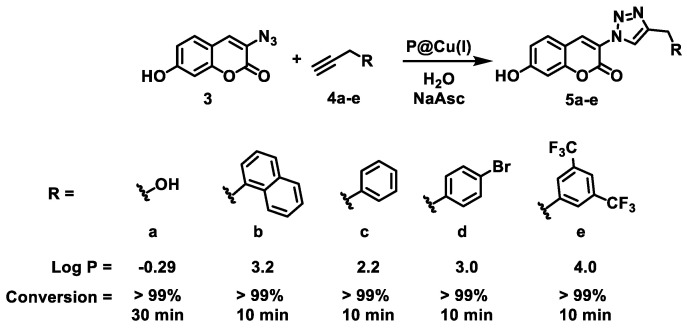
Chemical structures of coumarin azide **3** and alkynes **4a**−**e** in forming fluorescent products **5a**−**e**. We note that the reaction to **5a** remained homogeneous, whereas **5b**−**e** precipitated, leading to inhomogeneous mixtures.

To check the efficiency of the developed SCPNs towards CuAAC reactions, coumarin azide was chosen as the substrate to react with various hydrophilic and hydrophobic alkynes (Scheme 2) and hereby gain insights into the substrate scope of SCPNs in water. All SCPNs **P1**−**P3** have an average of 17 crosslinked ligands similar to 1 and, therefore, the same number of copper binding sites. First, **P1**−**P3** were formulated into SCPNs in water and mixed with CuSO_4_ in a 1:1 ligand:copper ratio to form **P1**−**P3@Cu(II)**. This ligand:copper ratio (1:1) was used as it is known that triazoles are weak binders of Cu(I) (advantageous for CuAAC). Multidentate interactions (from triazoles and the central donor nitrogen atom) are therefore required to have a high overall affinity for the metal, which is additionally stabilized by the carboxylate group [33]. The rate of the reaction between azide **3** and hydrophilic alkyne **4a** was first studied. Hereto, Cu(II) was reduced before the start of the reaction using 2 mM sodium ascorbate to form **P1**−**P3@Cu(I)**. The formation of fluorescent **5a** was followed by fluorescence spectroscopy over time and quantified using HPLC-UV. At 10 mol% Cu(I) concentration, the reactions with **P1**−**P3@Cu(I)** SCPNs were completed in 30 min. Despite differences in polymer microstructure, similar kinetics were observed (Figure 1A). On the other hand, control experiments using **P4@Cu(I)** or only Cu(I) did not proceed (Figure 1). Further, **P1@Cu(I)** was used to catalyze the click reaction with a series of more hydrophobic alkynes **4b**−**e**. In all cases, the reaction was completed within 10 min, owing to the high hydrophobicity of the alkynes that ensures accumulation near the catalytic active site in the hydrophobic domain.

**Figure 2:**
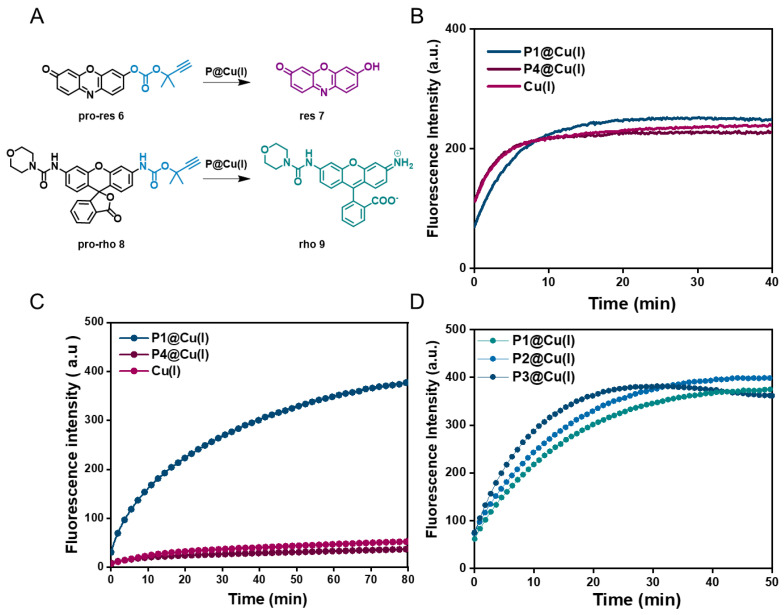
(**A**) Activation of pro-res **6** and pro-rho **8** to res **7** and rho **9**, respectively. Fluorescence kinetic profile of depropargylation reaction of substrates **6** and **8** using **P1@Cu(I)**, **P4@Cu(I)**, and Cu(I) in H_2_O; (**B**) activation of pro-res **6**, T = 20 °C; (**C**) activation of pro-rho **8**, T = 20 °C; (**D**) activation of pro-rho **8** using **P1**−**P3@Cu(I)**, T = 37 °C. In all cases, [Cu(I)] = 2 µM, [**6** and **8**] = 30 µM, [NaAsc] = 2 mM, [**P**] = ~120 nM. B: λ_ex_ = 532 nm and λ_em_ = 587 nm. C, D: λ_ex_ = 485 nm and λ_em_ = 520 nm.

To identify the optimal substrate that combines high reactivity in the presence of Cu(I) with the formation of a hydrophilic product that diffuses out of the hydrophobic pocket, we evaluated the efficacy of Cu(I) SCPNs in catalyzing the depropargylation reaction. For the depropargylation, substrates were designed with dimethyl propargyl as the protecting group to render the fluorophores non-fluorescent [12]. Single-molecule studies demand fluorophores with high brightness for a good signal-to-noise ratio and they also require good photostability. We selected rhodamine 110 and resorufin, which were previously used for single-molecule enzyme and heterogenous catalysis studies (Figure 2A) [19,20]. Pro-res **6** has dimethyl propargylic carbonate protection and pro-rho **8** has dimethyl propargylic carbamate protection based on morpholine carbonyl rhodamine 110 (rho **9**) (Figure 2A). The rate of pro-dye activation was monitored using fluorescence spectroscopy over time. Catalytically active **P1@Cu(I)**, control polymer **P4@Cu(I)**, and free Cu(I) were compared in each case to see the effect of the ligand on rate acceleration. Catalysis was performed at nanomolar concentrations of polymers (~120 nM, 0.02 mg/mL) in water and 30 µM substrate concentration, to assess their compatibility for single-molecule studies. At first, the activation of the substrate pro-res **6** was monitored. The fluorescence kinetic curves indicated that the reaction proceeded fast and the fluorescence intensity saturated within 10 min for all **P1@Cu(I)**, **P4@Cu(I)**, and Cu(I) (Figure 2B). Thus, the presence of the covalently attached ligand is not required for a fast turnover of the substrate. Also, substrate hydrolysis was observed in the presence of sodium ascorbate over a prolonged period of time (1 day). This implicated that pro-res **6** is not a good substrate for single-molecule studies as it can also become auto-hydrolyzed to the product and therefore cannot clearly bring out the heterogeneities in catalysis. 

On activating pro-rho **8**, **P1@Cu(I)** catalyzed the product formation significantly faster than **P4@Cu(I)** and Cu(I) (Figure 2C). Near quantitative conversion was obtained within 80 min at a 30 µM substrate concentration (Figure S14A). The complete consumption of the substrate was only observed at a higher temperature (37 °C) or at room temperature when the substrate and catalyst concentrations were increased to 100 µM and 10 µM, respectively (Figure S14). Therefore, we chose to perform further studies at 37 °C. A comparison of reaction kinetics at the ensemble level between all Cu(I) nanoparticles **P1**-**P3@Cu(I)** was then performed using pro-rho **8** as a substrate. For **P1**−**P3@Cu(I)** SCPNs, the differences in the polymer microstructure did not show significant effects on their catalytic activity (Figure 2D). They exhibited high catalytic efficiency which improved the chances to study and compare their kinetics at a single-particle level using single-molecule fluorescence spectroscopy. 

### 2.3. Cu(I) SCPNs Enzyme Kinetics—Michaelis–Menten Model

**Figure 3:**
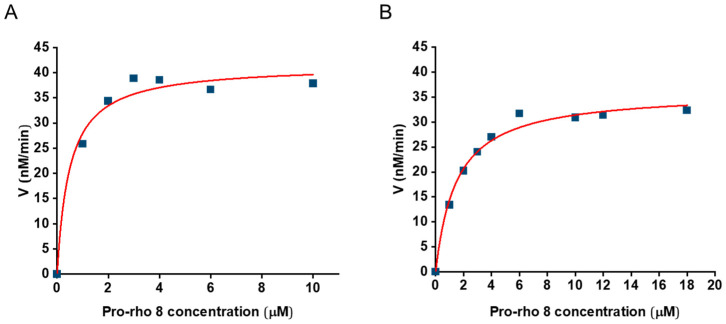
Michaelis–Menten fitting (y = (V_max_ × x)/(K_M_ + x) of reaction kinetics of pro-rho **8** activation by (**A**) **P1@Cu(I)**, values obtained for V_max_ = 41.51 ± 1.92 nM/min and K_M_ = 0.48 ± 0.14 µM, R^2^ = 0.97; (**B**) **P3@Cu(I)**, values obtained for V_max_ = 36.09 ± 1.11 nM/min and K_M_ = 1.49 ± 0.19 µM, R^2^ = 0.98. T = 37 °C, rate monitored using fluorescence kinetic experiment (t_0_ = 0 min and t_f_ = 10 min) and concentration of product determined from the calibration curve of rho **9**, [P] = 5 nM, in H_2_O. The squares in the curve represent the measured values of V at the given concentration of substrate.

It was previously reported that catalytic SCPNs exhibit enzyme-like kinetics following the Michaelis–Menten model [7,11]. Cu(I) SCPNs developed in this work were therefore evaluated for similar behavior under dilute conditions. For this, pro-rho **8** activation was monitored over time using **P1@Cu(I)** at 37 °C at 5 nM polymer concentration. The substrate concentration was increased from 100 to 800 nM, but no sign of saturation was observed. Upon further increasing the concentrations to micromolar concentration, the reaction rate started to saturate (Figure 3A). The saturation curve followed the Michaelis–Menten model and V_max_ was found to be 41.5 nM/min. Considering each particle as a single catalyst, from the equation V_max_ = *k*_cat_ [E] (here [E] = [P] = 5 nM), *k*_cat_ was found to be 498 h^−1^. To see if there are significant differences between the different polymer designs, **P3@Cu(I)** was also studied at substrate concentrations from 1 to 18 µM, and it also showed the same behavior (Figure 3B). Similarly, the *k*_cat_ was determined to be 433 h^−1^ for **P3@Cu(I)**. The results were fitted to the Lineweaver–Burk plot showing a linear relation between 1/V and 1/[S] (V = velocity or reaction rate; S = substrate concentration) (Figure S15). Zimmerman and co-workers previously reported the two-substrate enzyme kinetics behavior of an SCPN-based catalyst for CuAAC reactions (*V*_max_ = 5 µM/min) [7]. In this work, our main focus is on the depropargylation reaction to export the system to single-molecule studies. It is clear that SCPNs follow the one-substrate Michaelis–Menten model towards depropargylation reactions, albeit with a lower *V*_max_ of 36–41 nM/min.

### 2.4. Single-Particle Kinetics Using Single-Molecule Fluorescence Microscopy

Biotin-functionalized catalytic SCPNs based on **P1**−**P3** were immobilized on a plasma-treated coverslip according to a reported protocol [25]. For this, **P1**−**P3@Cu(II)** prepared in fresh MilliQ water was first mixed with streptavidin and then immobilized on a biotinylated BSA-covered coverslip. The coverslip was placed on a glass slide to create a capillary chamber of volume 30 μL. 

**Figure 4:**
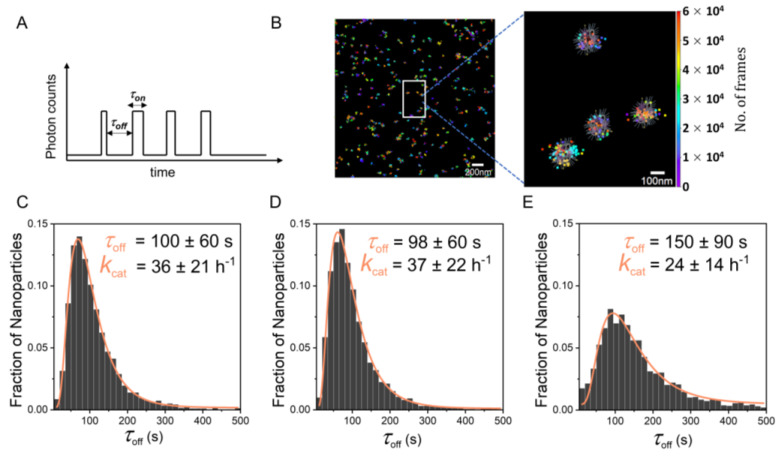
(**A**) Schematic single-molecule turnover trajectory showing off−on bursts of product formation. (**B**) Time-sequence single-molecule catalysis mapping of rho **9** formation by catalytic SCPNs, different colors indicate the different portions of product molecules formed sequentially in time (blue to red) after 30 k frames. Localized fluorescent bursts can be observed in the zoomed-in image, where the SCPN schematic is not to scale but represents the probability of products formed from individual SCPNs, after filtration of sparse localizations. (**C**) Distribution of *τ*_off_ of **P1@Cu(I)**. (**D**) Distribution of *τ*_off_ of **P2@Cu(I)**. (**E**) Distribution of *τ*_off_ of **P3@Cu(I)**. [**P**] = 1 nM, [pro-rho **8**] = 400 nM, [NaAsc] = 1 mM, T = 37 °C.

Single-particle kinetic studies were performed for catalytic nanoparticles **P1**−**P3@Cu(I)** using pro-rho **8** as substrate. Stochastic fluorescent bursts were recorded over time, which showed the localized formation of the product over time indicating single-particle catalysis (Figure 4A,B). The fluorescent time trajectory of these localized fluorescent bursts was recorded to obtain the waiting time *τ*_off_ between product formations. Note that *τ*_off_ is inversely proportional to the frequency of the events and therefore to the TOF. The distribution of the waiting time *τ*_off_ of individual nanoparticles was obtained from the kinetic measurements. All catalytic SCPNs **P1**−**P3@Cu(I)** evidently showed heterogeneity in catalysis at the single-particle level as observed from interparticle turnover dispersity (Figure 4C–E). Individual nanoparticles with high turnover as well as low turnover were detected in the same batch. We have previously mapped the polarity of individual SCPNs of similar polymer designs lacking the Cu-binding ligand using the NR-sPAINT technique. It was revealed that within a single batch of polymers, particles with higher or lower amounts of hydrophobic groups exist [25]. In this case, the origin of turnover dispersity within the same batch can be related to differences in the number of Cu(I) catalysts and its microenvironment per particle. Additionally, the particles with a higher number of hydrophobic grafts might exhibit a higher turnover frequency as they can better accommodate the hydrophobic substrate pro-rho **8**. 

To further compare different polymer designs, the distribution of *τ*_off_ was used to obtain the average *τ*_off_ value of each catalytic system **P1**−**P3@Cu(I)**. The inverse of the average *τ*_off_ was taken to obtain *k*_cat_, which ranged between 24 and 37 h^−1^ (Figure 4C–E) [34,35,36]. The *k*_cat_ derived from single-molecule studies (~30 h^−1^) is lower compared to that at the ensemble level (~400 h^−1^). Most likely, the immobilization of particles affects their dynamic nature partially. In addition, despite differences in polymer microstructure, all SCPNs showed similar *k*_cat_ values. It is likely that this is related to the reaction mechanism. While water acts as a nucleophile to induce cleavage of the dimethylpropargyl group (Figure S17), water is not involved in the rate-determining step of depropargylation reactions; therefore, the concentration of water or its diffusion to the hydrophobic pocket does not affect the rate of reaction [37]. Consequently, the small differences in the hydrophobic domains of different polymer designs may not contribute to significant differences in their catalytic activity. This was also as inferred from ensemble kinetic studies of pro-rho **8** activation (Figure 2D).

## 3. Discussion and Conclusions

Although there have been advances in the development of catalytic SCPNs for bio-orthogonal catalysis in biological settings, the effect of molar mass dispersity or stochastic incorporation of pendant groups on their catalytic efficiency has never been probed. This understanding is very valuable for the rational design of amphiphilic polymers that show high efficiency for future biomedical applications. To this end, we developed efficient catalytic Cu(I) SCPNs that can promote fast click and depropargylation reactions in water. We also designed and synthesized fluorogenic probes based on resorufin and rhodamine to tune the activity of catalytic SCPNs, and the best substrate pro-rho **8** was used for single-particle catalysis studies. We evaluated the catalytic behavior of SCPNs and found that they showed enzyme-like kinetics following the Michaelis–Menten model. This helped to calculate *k*_cat_ at the ensemble level which was found to be ~400 h^−1^. By careful optimization of the reaction conditions, we observed catalytic turnovers of individual particles by single-molecule kinetic measurements. The interparticle turnover dispersity was clearly evident from the distribution of the waiting time *τ*_off_. There were no significant differences between different polymer designs, which can be attributed to the similarity in the hydrophobic domains which accommodate the hydrophobic substrates. The effect of polymer microstructure on catalytic activity is not pronounced in Cu(I)-catalyzed reactions, as the turnover solely relies on the presence of Cu(I) in the vicinity of the chelating ligand, which is the same in all polymer designs. The turnover number (*k*_cat_) of all catalytic SCPNs **P1**−**P3@Cu(I)** during single-molecule kinetic experiments was found to be between 24 and 37 h^−1^, which is low compared to the values of around 400 h^−1^ obtained in ensemble measurements. We currently have no explanation for this discrepancy, which has not been observed in the case of single-enzyme studies [34]. Despite the low turnover numbers, we probed the diversity in reactivity of individual SCPNs for the first time. Although preliminary, our studies have laid a foundation for single-molecule SCPN kinetics studies which can be useful to probe changes in reactivity over time or in the presence of other biomolecules. In future, this approach can also be utilized for two-substrate systems where click reactions can be performed for the generation of CalFluors (Click-Activated Luminogenic Fluorophores) [38].

It is also important to consider the limitations of this approach during the discussed interpretations as there is the presence of a small fraction of auto-hydrolyzed products and also the possibility of products diffusing back to the hydrophobic pocket. Further improvements in the signal-to-noise ratio by developing more efficient catalytic systems with high turnovers similar to that of enzymes and better substrates can offer more reliable statistics. The current work is a small step towards unveiling the diversity of reactivity of individual catalytic SCPNs, which are hidden in ensemble measurements. In the future, this can help in the rational design of polymers by finding the relation between their defined three-dimensional structure and catalytic efficiency to attain enzyme-like selectivity and activity.

## 4. Materials and Methods

### 4.1. Materials and Instruments

All chemicals were purchased either from Merck or TCI chemicals. Poly(ethylene glycol) 2-aminoethyl ether biotin (average *M*_n_ = 2300 g/mol) was purchased from Sigma Aldrich. Deuterated solvents were purchased from Cambridge isotope laboratories. The dialysis membrane was regenerated cellulose tubing purchased from Spectra/Por^®^ with a molecular weight cut-off of 6–8 kDa. All solvents were purchased from Biosolve. The following polymers and compounds were synthesized according to previously developed protocols: ***p*PFPA_180_** [39], BTA amine [31], Gly **1**, diyne **2**, pro-rho **8**, rho **9**, 12-azidododecan-1-amine, and **P4** [12,26,29,40].

^1^H and ^13^C measurements were recorded on a Bruker 400 MHz spectrometer at ambient temperature, and chemical shifts were recorded concerning TMS as the internal standard for ^1^H and ^13^C spectra. Fluorescence measurements were performed on an Agilent Cary Eclipse fluorescence spectrophotometer using 1 cm × 1 cm pathlength quartz cuvettes. CD measurements were performed on a Jasco J-815 spectropolarimeter using 0.5 cm pathlength quartz cuvettes. Liquid chromatography–UV was performed using a Shimadzu UFLC-XR with a PDA detector with water +0.1% formic acid and ACN + 0.1% formic acid as eluents on a Kinetex column C18 5 mm EVO 100 Å. HPLC Method 1 for pro-dyes **6** and **8**: eluent A: water (0.1% formic acid); eluent B: acetonitrile (0.1% formic acid); and A/B = 90:10 isocratic 2 min, 90:10 to 0:100 in 2 min, isocratic 2 min, 0:100 to 90:0 in 2 min, and isocratic 2.0 min (flow = 0.2 mL/min). Size exclusion chromatography (SEC) measurements of poly(pentafluorophenyl)acrylate were recorded using a Shimadzu LC-2030C 3D with an RID-20 refractive index detector and a PDA detector at a flow rate of 1 mL min^−1^ with tetrahydrofuran as eluent with an Agilent mixed-C and mixed-D column in series at 40 °C. Exclusion limit = 2.000.000 g·mol^−1^, 7.5 mm i.d. × 300 mm. Calibration was performed using polystyrene standards from polymer laboratories. Dimethylformamide-SEC measurements of functionalized polymers were performed using a PL-GPC-50 plus (Varian Inc. Company, Palo Alto, CA, USA) equipped with a refractive index detector. DMF with 10 mM LiBr was used as an eluent at a flow rate of 1 mL min^−1^ on the Shodex GPC-KD-804 column at 50 °C. Exclusion limit = 100.000 Da, 0.8 cm i.d. × 300 mm, and it was calibrated using poly (ethylene oxide) from polymer laboratories. SEC measurements in PBS (pH = 7.4) were carried out on a Shimadzu CBM-20A System at 20 °C equipped with a Shimadzu RID-10A RI detector, SIL-20A autosampler, and 2 LC-20AD pumps on a Shodex OHpak SB-804 HQ column (exclusion limit = 1000 kDa, i.d. = 0.8 cm, and L = 300 mm) with a TSKgel SWXL type guard column (i.d. = 0.6 cm; L = 40 mm), with PBS as an eluent at a constant flow rate of 0.8 mL min-1. The column was calibrated against poly(ethylene oxide) (PEO) (Polymer Laboratories). Dynamic light scattering experiments were performed using a Malvern Zetasizer with an 830 nm laser and an angle of scattering 90°. Single-molecule images were obtained in an Oxford Nanoimager microscope (ONI, Oxford, UK).

### 4.2. Pro-Dye and Polymer Synthesis

#### 4.2.1. Pro-Res **6** (2-Methylbut-3-yn-2-yl (3-oxo-3H-Phenoxazin-7-yl) Carbonate) 

Triphosgene (445 mg, 1.5 mmol) was dissolved in 2 mL dichloromethane under argon atmosphere and was cooled in an ice bath. 2-Methylbut-3-yn-2-ol (290 µL, 3 mmol) and pyridine (266 µL, 3.3 mmol) were added dropwise to the above solution of triphosgene, which resulted in a white precipitate. The mixture was allowed to stir for 1.5 h in an ice bath, followed by the evaporation of dichloromethane under argon flow. To this crude mixture, 3 mL dimethylformamide was added, resulting in a clear solution. This solution was added dropwise into another flask containing resorufin (160 mg, 0.7 mmol) and triethylamine (200 µL, 1.5 mmol) dissolved in 16 mL dimethylformamide under an argon atmosphere in an ice bath. After the addition, the mixture was allowed to stir in an ice bath for 15 min and then at room temperature for 2 h. After monitoring the reaction by thin layer chromatography (TLC), the mixture was decanted to remove unreacted resorufin. To the mixture, 200 mL water was added and then was extracted using dichloromethane (4 × 20 mL). The combined organic phase was washed with brine solution and dried over sodium sulfate and dichloromethane was evaporated under reduced pressure at room temperature. The product was then dried under vacuum and purified by column chromatography on silica gel (EtOAc/heptane—30/70 *v*/*v*) to obtain the pure product as a brownish powder.

Yield = 15 mg, 6%. ^1^H NMR (400 MHz, CDCl_3_) δ 7.80 (d, *J* = 8.7 Hz, 1H), 7.43 (d, *J* = 9.8 Hz, 1H), 7.29 (s, 1H), 7.23 (d, *J* = 2.5 Hz, 1H), 6.87 (dd, *J* = 9.8, 2.0 Hz, 1H), 6.33 (d, *J* = 2.0 Hz, 1H), 2.65 (s, 1H), 1.82 (s, 6H). ^13^C NMR (100 MHz, CDCl_3_) δ 144.34, 135.22, 134.80, 131.15, 118.64, 109.13, 107.30, 83.07, 77.21, 76.05, 73.92, 28.65. FT-IR (ATR): *v* (cm^−1^) 3290, 3048, 2994, 1772, 1623, 1608, 1572, 1515, 1246, 1220, 1196, 1129, 885, 848, 772, 667. MALDI-TOF-MS: m/z calc: 323.0; found: 323 [M]^−^, deprotected product resorufin 212 [M − H]^−^.

#### 4.2.2. Post-Functionalization of ***p***-**PFPA_180_** to **P1_a_**–**P3_a_**

**P1_a_**: ***p***-**PFPA_180_** (172 mg, 1 eq, 0.0040 mmol) was dissolved in dry tetrahydrofuran in a Schlenk flask and was kept in a preheated oil bath at 50 °C. To this solution, 12-azidododecan-1-amine (32 mg, 36 eq, 0.14 mmol) was added and stirred for 2 h. Incorporation was followed by ^19^F NMR by comparing the peaks of free pentafluorophenol with those in the polymer backbone. Afterwards, biotinylated PEG amine (55 mg, 6 eq, 0.024 mmol) was added to the mixture and stirred for 2 h, and the incorporation was monitored. Finally, pre-dried Jeffamine@1000 (800 mg, 212 eq, 0.8 mmol) was added. The reaction mixture was then left overnight under argon and the completion of the reaction was again monitored using ^19^F NMR. Then, the reaction mixture was purified by dialysis (1 × 1 L methanol, 2 × 1 L tetrahydrofuran) for 3 days. After dialysis, tetrahydrofuran was reduced to ~3 mL using a rotary evaporator and the polymer was precipitated into ice-cold pentane (800 mL). The polymer was dried under vacuum overnight at 50 °C to yield a colorless solid and was stored at −19 °C.

Yield: 380 mg, *M*_theoretical_ = 161 kD, *M*_n, SEC-DMF_ = 59.1 kD, *Ð* = 1.21. ^1^H NMR (400 MHz, CDCl_3_) δ 6.92–6.01 (br), 4.15–3.90 (br), 3.84–3.14 (m), 1.90–1.82 (br), 1.72–1.52 (s), 1.41–0.93 (m). FT-IR (ATR): *v*(cm^−1^) 3520.56, 2865.86, 2096.31, 1650.15, 1544.45, 1454.07, 1348.63, 1325.4, 1296.42, 1249.42, 1096.62, 947.46, 848.32, 522.91. *M*_theoretical_ = 161 kD, *M*_n, SEC-DMF_ = 59.1 kD, *Ð* = 1.21.

*The same protocol was followed for all polymers with varying ligands as specified below.*

**P2_a_**: ***p***-**PFPA_180_** (180 mg, 1 eq, 0.0042 mmol), 12-azidododecan-1-amine (34 mg, 36 eq, 0,15 mmol), BTA amine (25 mg, 9 eq, 0.037 mmol), biotinylated PEG amine (57 mg, 6 eq, 0.025 mmol), and Jeffamine@1000 (890 mg, 212 eq, 0.8 mmol). The polymer was dried under vacuum at 50 °C to yield a colorless solid and was stored at −19 °C. ^1^H NMR (400 MHz, CDCl_3_) δ 8.51–8.39 (br), 6.86–6.32 (br), 4.18–3.13 (m), 1.91–1.51 (m), 1.41–1.04 (m). FT-IR (ATR): *v* (cm^−1^) 3519.56, 2866.02, 2096.51, 1650.73, 1543.94, 1454.53, 1348.76, 1325.35, 1294.99, 1249.98, 1094.69, 947.35, 848.13, 523.42. *M*_theoretical_ = 158 kD, *M*_n, SEC-DMF_ = 44.3 kD, *Ð* = 1.20.
**P3_a_**: ***p***-**PFPA_180_** (150 mg, 1 eq, 0.0031 mmol), 12-azidododecan-1-amine (25 mg, 36 eq, 0,11 mmol), dodecyl amine (15.4 mg, 27 eq, 0.083 mmol), biotinylated PEG amine (47 mg, 6 eq, 0.018 mmol), and Jeffamine@1000 (657 mg, 212 eq, 0.65 mmol). The polymer was dried under vacuum at 50 °C to yield a colorless solid and was stored at −19 °C. ^1^H NMR (400 MHz, CDCl_3_) δ 6.86–6.13 (br), 4.27–3.95 (br), 3.87–3.13 (m), 1.94–0.82 (m). FT-IR (ATR): *v* (cm^−1^): 3301.87, 2863.67, 2096.15, 1649.47, 1540.03, 1454.47, 1346.79, 1324.91, 1295.06, 1249.38, 1199.43, 1098.22, 1039.84, 947.47, 845.32, 522.96. *M*_theoretical_ = 165 kD, *M*_n,SEC-DMF_ = 47.5 kD, *Ð* = 1.25.

#### 4.2.3. Incorporation of the diyne **2** to **P1**–**P3**

Diyne **2** was incorporated into the polymeric backbone according to the modified ‘folding and cross-linking’ strategy reported by Zimmerman and co-workers [6]. In a 25 mL round bottom flask, **P1_a_** (100 mg, 0.00062 mmol, 1 eq) was dissolved in 5 mL water. The solution was sonicated for 1 h. The azide pendant groups in the polymer backbone are present in 30 eq (considering the incorporation ratio monitored from ^19^F NMR). Therefore, diyne **2** (1.7 mg, 0.011 mmol, 18 eq; where 15 eq azide was still left, equivalence optimized by the stepwise increase of diyne **2**) was added to this solution of nanoparticles in water, followed by the addition of CuSO_4_ (1 mg, 0.006 mmol, 10 eq) and sodium ascorbate (20 mg, 0.10 mmol, 161 eq). The reaction mixture was then allowed to stir overnight under argon. Afterwards, 1 g Chelex resin was added to the reaction mixture to remove copper and was allowed to stir for another 24 h. Followingly, the resin was removed by filtration and the solution was dialyzed in deionized water (5 × 1 L) for 5 days. Afterwards, water was slowly and carefully removed using the rotary evaporator and the polymer was dried under vacuum overnight at 50 °C to obtain a pale-yellow solid in all cases. Yield = 20–30 mg. The same procedure with the same amounts was followed for **P2** and **P3**.

**P1**: ^1^H NMR (400 MHz, D_2_O) δ 8.45–8.40 (br), 8.22–8.16 (m), 4.85–4.81 (d), 3.91–3.27 (m), 2.12–1.76 (s), 1.27–0.95 (br). FT-IR (ATR): *v* (cm^−1^): 3504.07, 2866.26, 1648.32, 1542.17, 1453.42, 1348.53, 1297.19, 1249.3, 1094.89, 946.76, 845.98. *M*_theoretical_ = 164 kD, *M*_n, SEC-PBS_ = 15.5 kD, *Ð* = 1.38.
**P2**: ^1^H NMR (400 MHz, D_2_O) δ 8.47–8.43 (br), 8.23–8.14 (m), 4.85–4.81 (s), 3.81–3.29 (m), 1.31–0.99 (br). FT-IR (ATR): *v* (cm^−1^): 3437.19, 2867.07, 1647.18, 1544.54, 1452.73, 1348.65, 1294.81, 1249.96, 1094.27, 947.04, 846.59, 521.88. *M*_theoretical_ = 161 kD, *M*_n, SEC-PBS_ = 23.4 kD, *Ð* = 1.35.
**P3**: ^1^H NMR (400 MHz, D_2_O) δ 8.41–8.37 (br), 8.22–8.16 (m), 4.85–4.81 (s), 4.53–4.35 (s), 3.92–3.27 (m), 1.36–1.12 (br). FT-IR (ATR): *v* (cm^−1^): 3436.86, 2869.53, 1646.34, 1548.24, 1454.48, 1348.8, 1296.07, 1250.18, 1091.99, 947.74, 844.89, 805.17, 523.09. *M*_theoretical_ = 168 kD, *M*_n, SEC-PBS_ = 16.3 kD, *Ð* = 1.35.

### 4.3. Ensemble Catalysis Measurements

All substrate stock solutions were prepared in DMSO: coumarin azide **3** at 100 mM, and all alkynes **4a**–**e** at 400 mM concentration. Sodium ascorbate stock solution was prepared in MilliQ water at 200 mM and CuSO_4_ was also prepared in MilliQ water at a 10 mM concentration. **P1**–**P3** were prepared by dissolving 1 mg polymer in 991 µL MilliQ water by sonicating for 1 h. The samples were allowed to rest for 1 h, and then 9 µL of CuSO_4_ stock solution was added into the solution and stirred for 15 min to reach a final concentration of ~6 µM of **P1**–**P3@Cu(II)** and 90 µM **Cu(II)** concentration in the sample (ligand:Cu molar ratio = 1:1).

For CuAAC reactions with polymers, 333.3 µL of the **P1**–**P3@Cu(II)** stock solution (as prepared above) was added to a 10 mm fluorescence cuvette, followed by the addition of 2630.7 µL of MilliQ water. To this solution, 3 µL of coumarin azide **3** stock solution and 3 µL of alkyne stock solution **4a**–**e** (depending on each reaction) was added. Finally, 30 µL of sodium ascorbate stock solution was added just before the start of the reaction to reduce Cu(II) to Cu(I) and the reaction was monitored by fluorescence spectroscopy at λ_ex_ = 340 nm and λ_em_ = 470 nm over time with stirring. For hydrophilic alkyne **5a**, the progress of the reaction was followed by an increase in fluorescence intensity of the product formed over time. After 30 min, when fluorescence intensity reached a plateau, an aliquot was taken from the reaction mixture and injected to HPLC-UV. For hydrophobic alkynes **5b**–**e**, the fluorescence intensity increased for up to 3 min, after which it started decreasing. In the case of hydrophobic alkynes, the product formed was more hydrophobic, making the solution non-homogenous and making it difficult to track the reaction progress using fluorescence spectroscopy. Therefore, aliquots from the reaction mixture were dissolved in acetonitrile to reach a water:ACN ratio of 1:1 and this was injected to HPLC-UV. In all cases, products **5b**–**e** were found to be the major fraction but the complete conversion was only obtained in 10 min. For CuAAC control reactions, the same procedure as above was followed where at first 90 µM of the CuSO_4_ solution was prepared without polymers.

### 4.4. Depropargylation Reactions

All substrate stock solutions **6** and **8** were prepared in DMSO at a 30 mM concentration. Sodium ascorbate stock solution was prepared in MilliQ water at 200 mM and CuSO_4_ was also prepared in MilliQ water at a 10 mM concentration. **P1**–**P3** and **P4** were prepared by dissolving 1 mg polymer in 991 µL MilliQ water by sonicating for 1 h. The samples were allowed to rest for 1 h, and then 9 µL of the CuSO_4_ stock solution was added into the solution and stirred for 15 min to reach a final concentration of ~6 µM of **P1**–**P4@Cu(II)** and 90 µM **Cu(II)** concentration in the sample (ligand:Cu molar ratio = 1:1).

For the depropargylation reaction at a 30 µM substrate concentration, 66.6 µL of **P1**-**P4@Cu(II)** stock solution was added to a 10 mm fluorescence cuvette, followed by the addition of 2900.4 µL of MilliQ water. To this solution, 3 µL of substrate **6** or **8** stock solution was added. Finally, 30 µL of sodium ascorbate stock solution was added just before the start of the reaction to reduce Cu(II) to Cu(I) and the reaction was monitored by fluorescence spectroscopy at λ_ex_ = 532 nm and λ_em_ = 587 nm for substrate **6** and λ_ex_ = 485 nm and λ_em_ = 520 nm for substrate **8**. Aliquots from the reaction mixture were taken at specified time intervals and diluted with acetonitrile (water:ACN = 1:1 *v*/*v*) and injected to HPLC-UV to confirm the formation of the products. The reactions were performed either at room temperature or at 37 °C as specified. 

For the depropargylation reaction at the 100 µM substrate pro-rho **8** concentration, a 100 mM stock solution of **8** was made in DMSO. An amount of 333.3 µL of the **P1@Cu(II)** stock solution was added to a 10 mm fluorescence cuvette, followed by the addition of 2633.7 µL of MilliQ water. To this solution, 3 µL of substrate **8** stock solution was added. Finally, 30 µL of sodium ascorbate stock solution was added just before the start of the reaction to reduce Cu(II) to Cu(I) and the reaction was monitored by fluorescence spectroscopy at λ_ex_ = 485 nm and λ_em_ = 520 nm. Aliquots from the reaction mixture were taken at specified time intervals and diluted with acetonitrile (water:ACN = 1:1 *v*/*v*) and injected to HPLC-UV to confirm the formation of the products. For control reactions, the same procedure as above was followed where at first 90 µM of CuSO_4_ solution was prepared without polymers.

### 4.5. Michaelis–Menten Kinetics

Calibration of rho **9**: The product rho **9** calibration curve was prepared from a **9** concentration of 100 to 600 nM at λ_ex_ = 485 nm and λ_em_ = 520 nm using the fluorescence spectrophotometer. For this, 100 µM stock solution of rho **9** was prepared in MilliQ water to minimize the presence of DMSO in the reaction mixture. From the calibration curve, the concentration of the product formed in the reaction mixture over time was interpolated. 

Sample preparation: **P1**–**P3@Cu(II)** stock solution was prepared by dissolving 1 mg **P1**–**P3** in 982 µL MilliQ water by sonicating for 1 h. The samples were allowed to rest for 1 h, followed by the addition of 18 µL CuSO_4_ stock solution and stirring for 15 min. The ligand:Cu ratio was 1:2 in this case, in order to compare the *k*_cat_ with single-molecule kinetic studies where the above-mentioned ratio was required to obtain sufficient turnovers. This leads to a final concentration of the polymer of ~ 6 µM and 180 µM Cu(II) in the sample. An amount of 100 µM pro-rho **8** stock solution was freshly prepared in MilliQ water by diluting it from 10 mM DMSO stock solution (1 *v*/*v*% DMSO in water), to minimize the amount of DMSO in the reaction mixture while ensuring the solubility of pro-rho **8** in water. An amount of 200 mM of the stock solution of sodium ascorbate was used.

Kinetic studies of **P1@Cu(I)**: 3 µL of **P1@Cu(I)** stock solution was added to a 10 mm fluorescence cuvette, followed by the addition of MilliQ water (2979 µL–2442 µL, depending on the increasing amount of substrate concentration keeping the total volume 3 mL). To this solution, 3 µL–540 µL of pro-rho **8** stock solution (100 µM in water) was added. After mixing, the fluorescence cuvette was placed in the spectrophotometer with stirring. Just before the start of the kinetic measurements, 15 µL of sodium ascorbate stock solution was added (to reach 1 mM concentration in the reaction mixture to keep it the same as that used for single-molecule studies). The initial rate of the reaction was monitored by the change in the fluorescent intensity in the first 10 min after the start of the experiment. The rate of change in concentration was calculated from the calibration curve of rho **9**. The rate of change in concentration was then plotted with the change in substrate concentration, where the rate was found to saturate with increasing substrate concentration following the Michaelis–Menten model, which was fitted to obtain the maximum velocity or rate *V*_max_. From the *V*_max_, the *k*_cat_ was found by following the equation *V*_max_ = *k*_cat_[E], where [E] is the enzyme concentration, and in this case, the polymer concentration is approximately 5 nM. Therefore, from the Michaelis–Menten model fitted for **P1@Cu(I)** kinetics, we found that *V*_max_ = 41.5 nM/min and the corresponding *k*_cat_ was calculated to be 8.3 min^−1^ or 498 h^−1^(*V*_max_/[P = 5 nM]).

### 4.6. Single-Particle Kinetic Studies

Coverslips (24 × 24 mm^2^) immersed in methanol were kept in the ultrasound sonication bath for 15 min. Followingly, they were dried with nitrogen flow and plasma-treated for 1 min. The coverslip was kept over the glass slide using two strips of double-sided tape to form a capillary chamber with an inner volume of ~30 µL. Then, 30 μL of 0.1 mg/mL biotinylated bovine serum albumin dissolved in buffer A (10 mM TRIS-HCl, 50 nM NaCl, pH 8.0) was introduced into the chamber and incubated for 1 h. Afterwards, the unbound BSA was washed away by flushing the chamber with 200 µL Buffer A solution. An amount of 5 µM of **P1**–**P3@Cu(II)** biotinylated SCPNs was mixed with 0.03 mg/mL streptavidin. This solution was then shaken at 300 rps at room temperature for 45 min. This solution was then diluted to reach desired concentration (5 nM–12.5 nM) as specified for each measurement. Diluted SCPNs were then introduced into the chamber and incubated for 45 min. The unbound particles were then washed again using 100 µL of MilliQ water. 

A 100 µM stock solution of substrate pro-rho **8** was prepared in MilliQ water by diluting it from a 10 mM stock solution in DMSO. An amount of 200 mM stock solution of sodium ascorbate was also prepared. For catalysis studies, substrate pro-rho **8** and sodium ascorbate were mixed together to reach the specified concentration. For the final kinetic studies, 400 nM substrate pro-rho **8** to 1 mM sodium ascorbate was used. To reach this, the 100 µM stock solution was first diluted to 10 µM concentration in water. An amount of 40 µL of 10 µM pro-rho **8** stock solution was added to 955 µL MilliQ water, followed by the addition of 5 µL of 200 mM sodium ascorbate stock solution. The pro-dye was freshly purified before single-molecule catalysis studies and sample preparation was performed just before single-molecule measurements to minimize the presence of the auto-hydrolyzed product. 

The 30 µL of the prepared substrate–ascorbate solution was then introduced into the chamber and the glass slide was introduced on an ONI microscope with the temperature preset at 37 °C. The sample was illuminated with a 480 nm laser, using total internal reflection fluorescence (TIRF) for 60,000 frames, and dye fluorescence was recorded using a 100×, 1.4 NA oil immersion objective, passed through a beam splitter. Images were acquired on a 427 × 520 pixel region (pixel size 0.117 μm) of a sCMOS camera, with an exposure time of 20 ms. Images were reconstructed using the ONI Nimos software (https://oni.bio/) in order to identify and fit the point spread functions and obtain the super-resolved position of the target molecule. 

## Figures and Tables

**Table 1:** Overview of the copolymer composition (a–e), number-average molecular weight (*M*_n_), and molar mass dispersity (*Đ*) of ***p*PFPA_180_** and **P1**−**P4**. Hydrodynamic radius (*R*_H_) of nanoparticles before (**P1a**−**P3a**) and after incorporation of the ligand (**P1**−**P3**) and **P4** in H_2_O.

Polymer	a	b	c	d	e	*Ð*	*M*_n, SEC_ (kDa)	*M*_n, theoretical_ (kDa)	*R*_H_ (nm)
***p*** **PFPA_180_**						1.19 ^x^	30.2 ^x^	42	-
**P1a**	3	18	-	-	71	1.21 ^y^	59.1 ^y^	161	4.3
**P2a**	3	18	-	5	69	1.20 ^y^	44.3 ^y^	158	5.0
**P3a**	3	18	12	-	67	1.25 ^y^	47.5 ^y^	165	5.0
**P1**	3	18	-	-	71	1.38 ^z^	15.5 ^z^	164	3.4
**P2**	3	18	-	5	69	1.35 ^z^	23.4 ^z^	161	4.9
**P3**	3	18	12	-	67	1.35 ^z^	16.3 ^z^	168	4.8
**P4**	-	-	20	-	80	1.16 ^y^	24.4 ^y^	181.0	6.0

The values for the composition of the polymers a-e were determined by ^19^F NMR. *M*_n_ and *Ð* were measured by (x) SEC in THF relative to poly(styrene) standards, (y) SEC in DMF with 10 mM LiBr relative to poly(ethylene oxide) standards, and (z) SEC in PBS relative to poly(ethylene oxide) standards. Some hydrolysis of the pentafluorophenylacrylate backbone was observed during post-functionalization with amines and was quantified as **P1a** = 8% and **P2a** = 5%.

## Data Availability

All data are available from the corresponding author upon request.

## Supplementary Materials

The following supporting information can be downloaded at https://www.mdpi.com/article/10.3390/molecules29081850/s1, Scheme S1: Structure of polymer P4; Figures S1–S7: NMR spectra; Figures S8–S12: IR spectroscopy, SEC chromatography, CD spectroscopy and DLs measurements; Figures S13 and S14: HPLC-UV; Figure S15: Ensemble kinetics studies; Figure S16: Single-particle kinetic studies; Figure S17: Reaction mechanism of Cu(I) catalyzed depropargylation reaction.
