# Supplementary material for: Effect of Particle Heterogeneity in Catalytic Copper-Containing Single-Chain Polymeric Nanoparticles Revealed by Single-Particle Kinetics
Source: Molecules. 2024 Apr 18;29(8):1850. doi: 10.3390/molecules29081850 (PMC11054931; doi:10.3390/molecules29081850)
Supplement: Supplementary file 1 [file molecules-29-01850-s001.zip › molecules-2953252-supplementary.pdf]

1. Structure of polymer P4

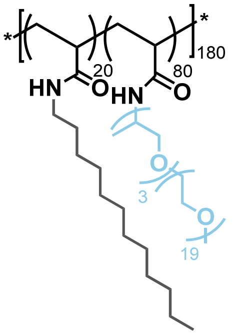

Scheme S1: Structure of polymer **P4**

2. NMR Spectra

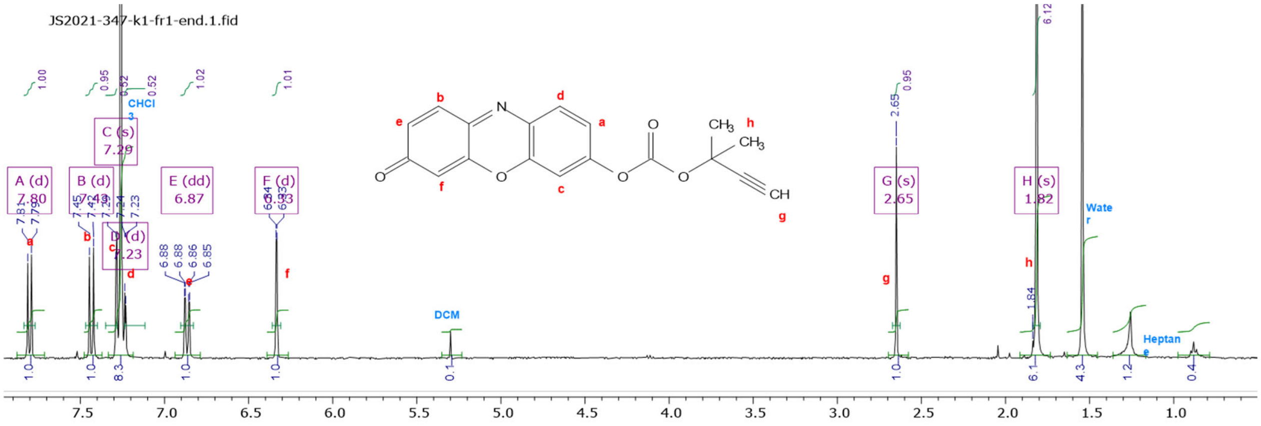

Figure S1: ^{1}H NMR spectrum of pro-res **6**

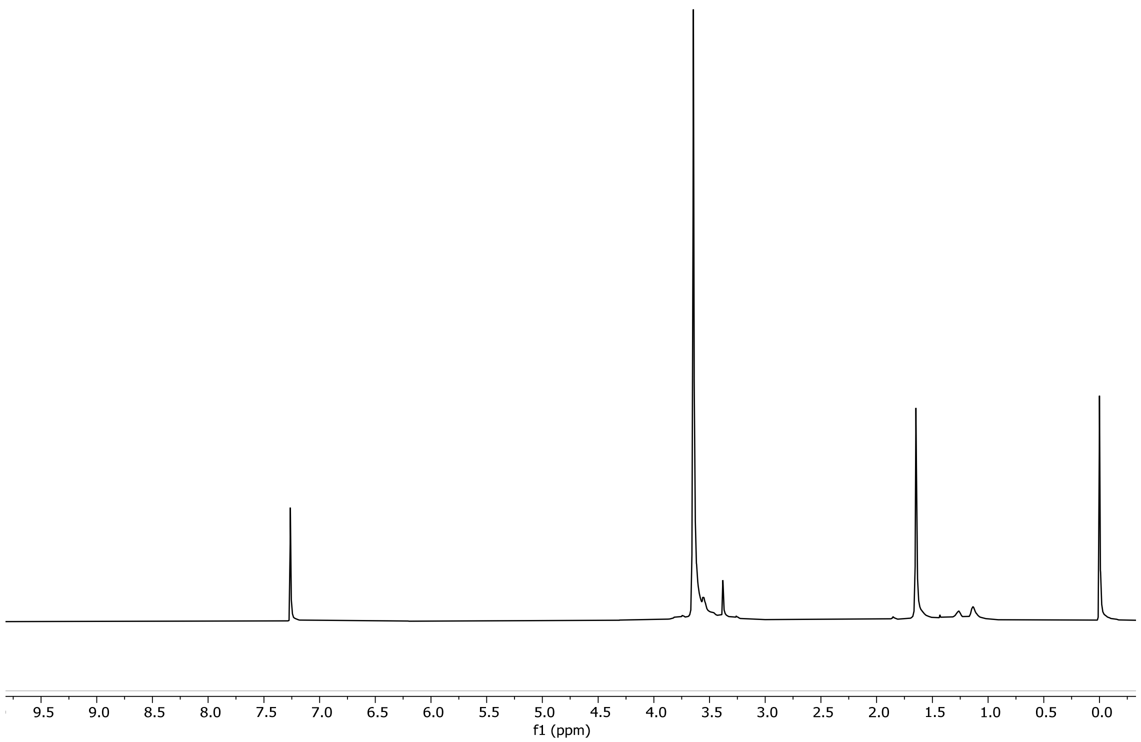

Figure S2: ^{1}H NMR spectrum of **P1** in $CDCl_3$.

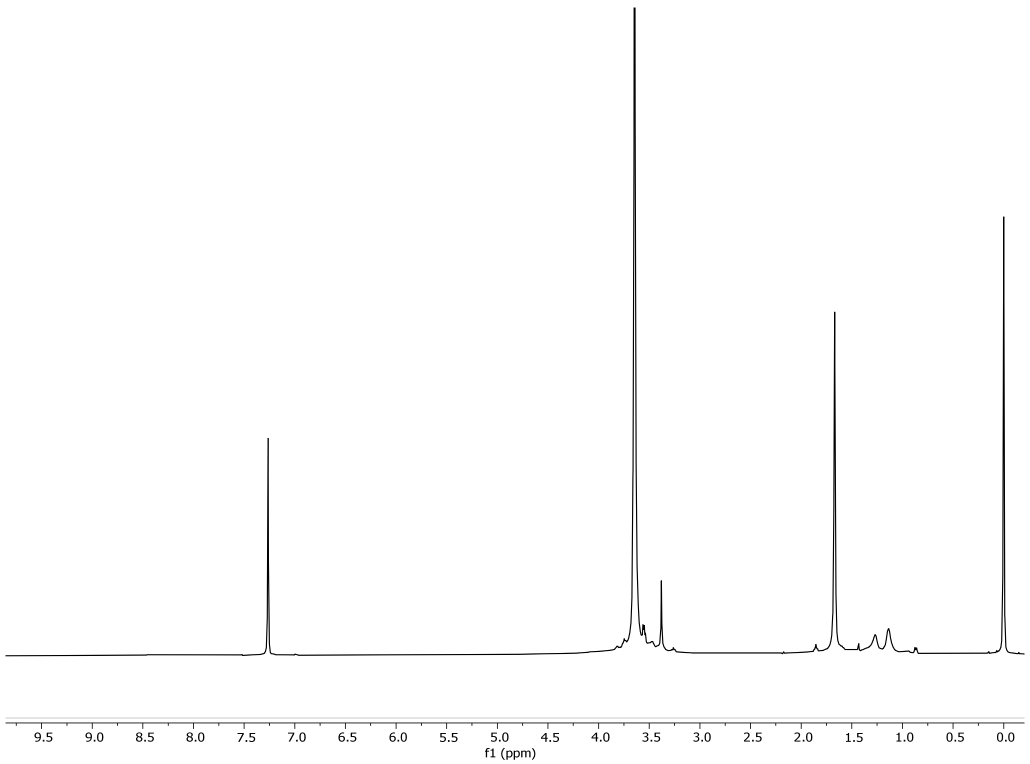

Figure S3: ^{1}H NMR spectrum of **P2** in $CDCl_3$.

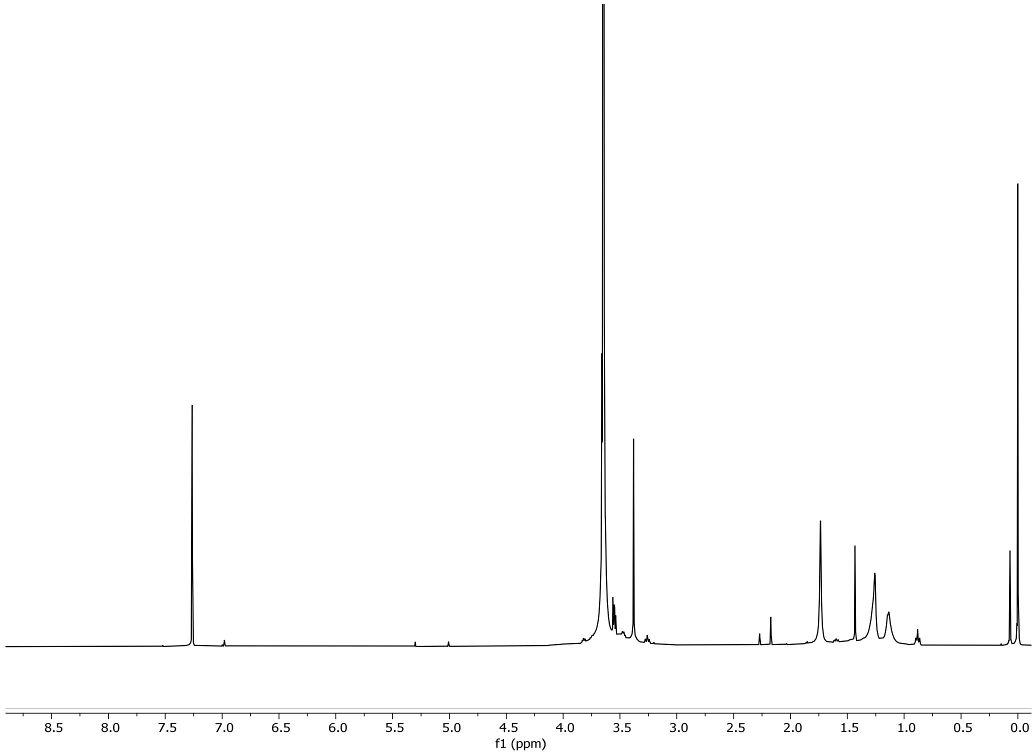

Figure S4: ^{1}H NMR spectrum of **P3** in $CDCl_3$.

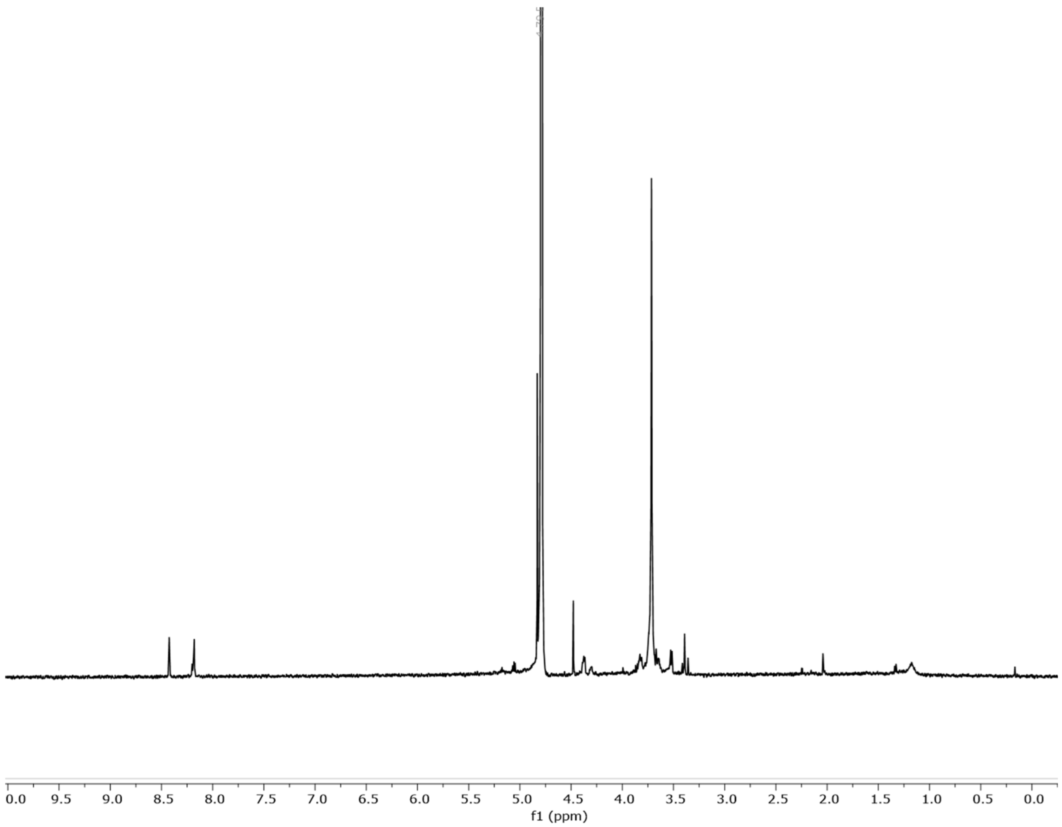

Figure S5: ^{1}H NMR spectrum of **P1a** in D_2O.

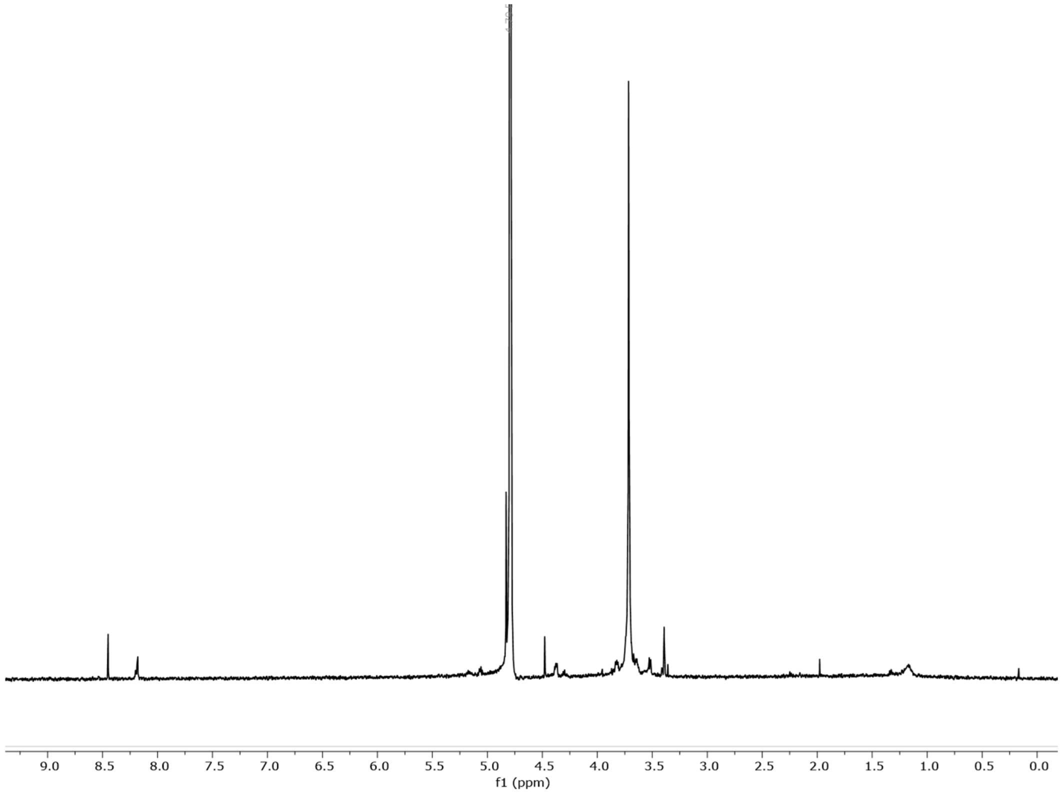

Figure S6: ^{1}H NMR spectrum of **P2a** in D_2O.

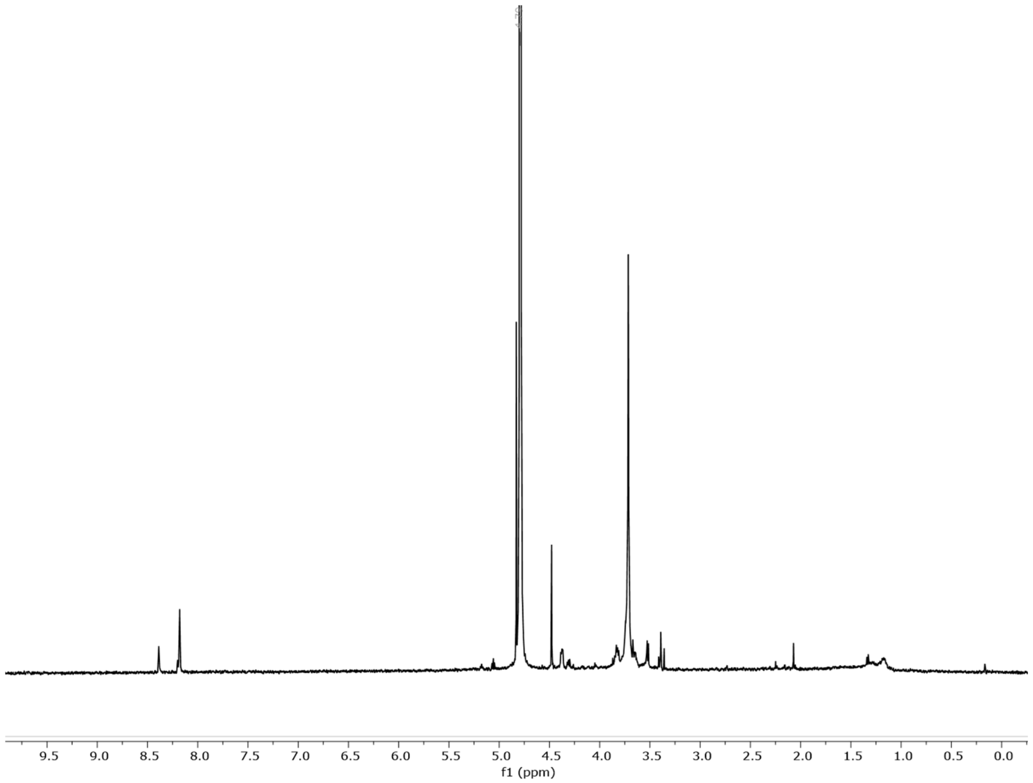

Figure S7: ^{1}H NMR spectrum of **P3a** in D_2O.

3. IR spectroscopy

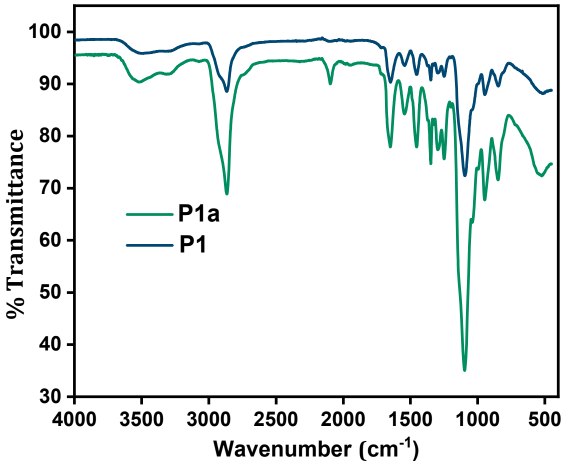

Figure S8: Infrared spectra of amphiphilic polymer of **P1$_a$** and **P1**: before and after incorporation of diyne **2** confirming the disappearance of azide peak at 2096 cm^{-1}.

4. SEC chromatography

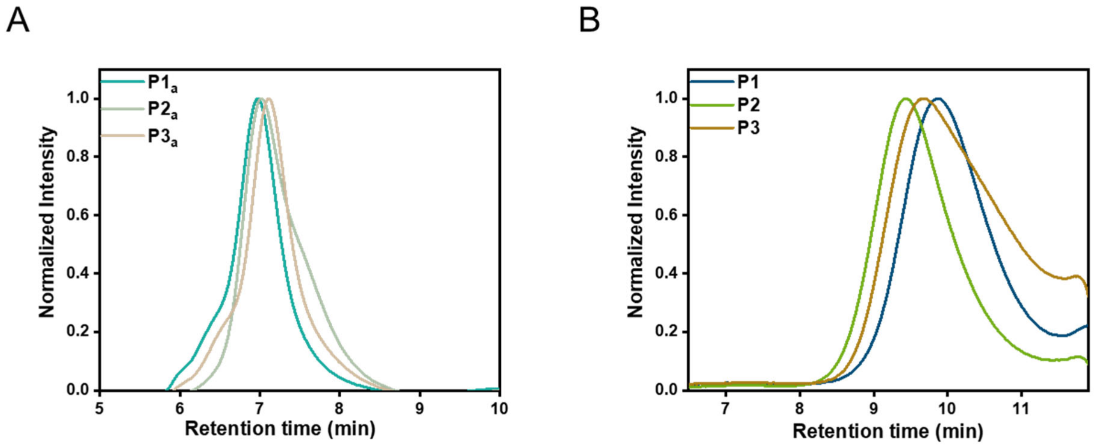

Figure S9: A) Normalised SEC traces in DMF of polymer precursors **P1$_a$-P3$_a$** (1 mg/mL). B) Normalised SEC traces in PBS of **P1-P3** after incorporation of ligand (1 mg/mL).

5. CD Spectroscopy

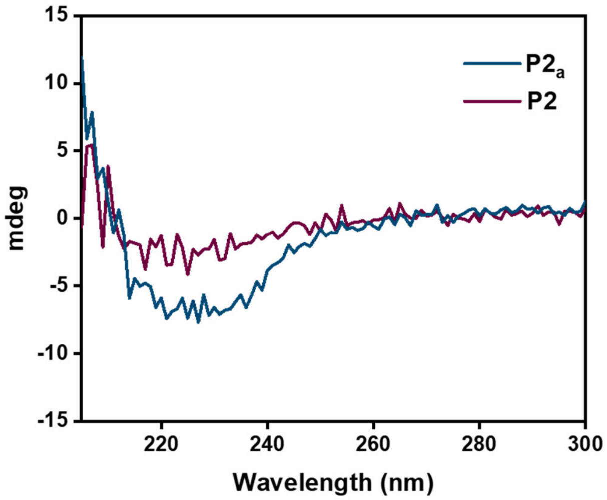

Figure S10: Circular dichroism spectra of polymeric nanoparticles: **P2$_a$** and **P2** after intramolecular crosslinking, [**P**] = 0.5 mg/mL, T = 20 °C, in H_2O: The self-assembly of BTA grafts before and after the incorporation of the crosslinking ligands showed a negative CD signal centred around λ = 225 nm indicating the presence of left-handed (M) helical BTA aggregates. The incorporation of the ligand reduced the size of the CD effect, indicating that the degree of BTA self-assembly was negatively affected.

6. Dynamic light scattering measurements

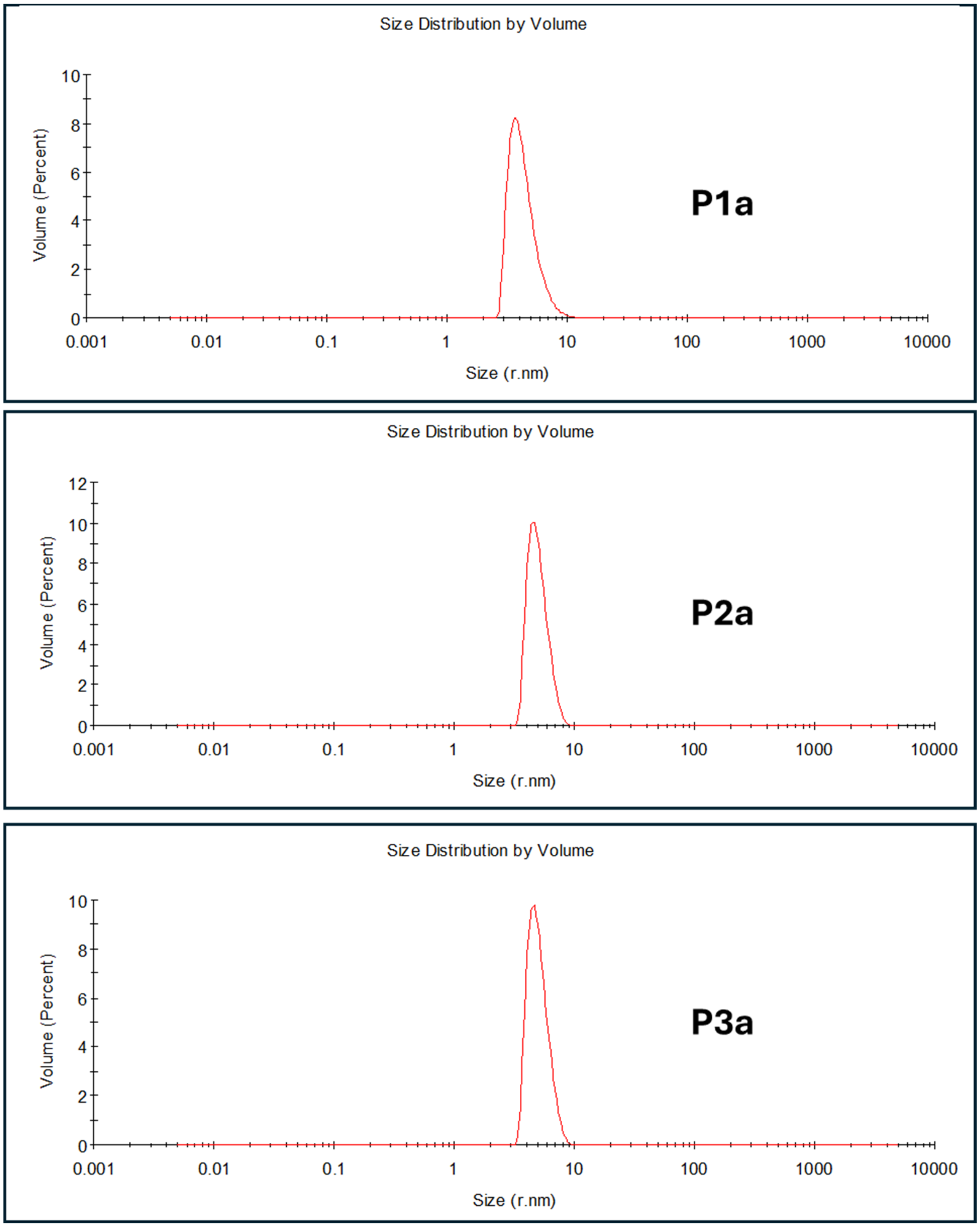

Figure S11: Dynamic light scattering measurements of **P1a-P3a**, [**P**] = 1 mg/mL in H_2O.

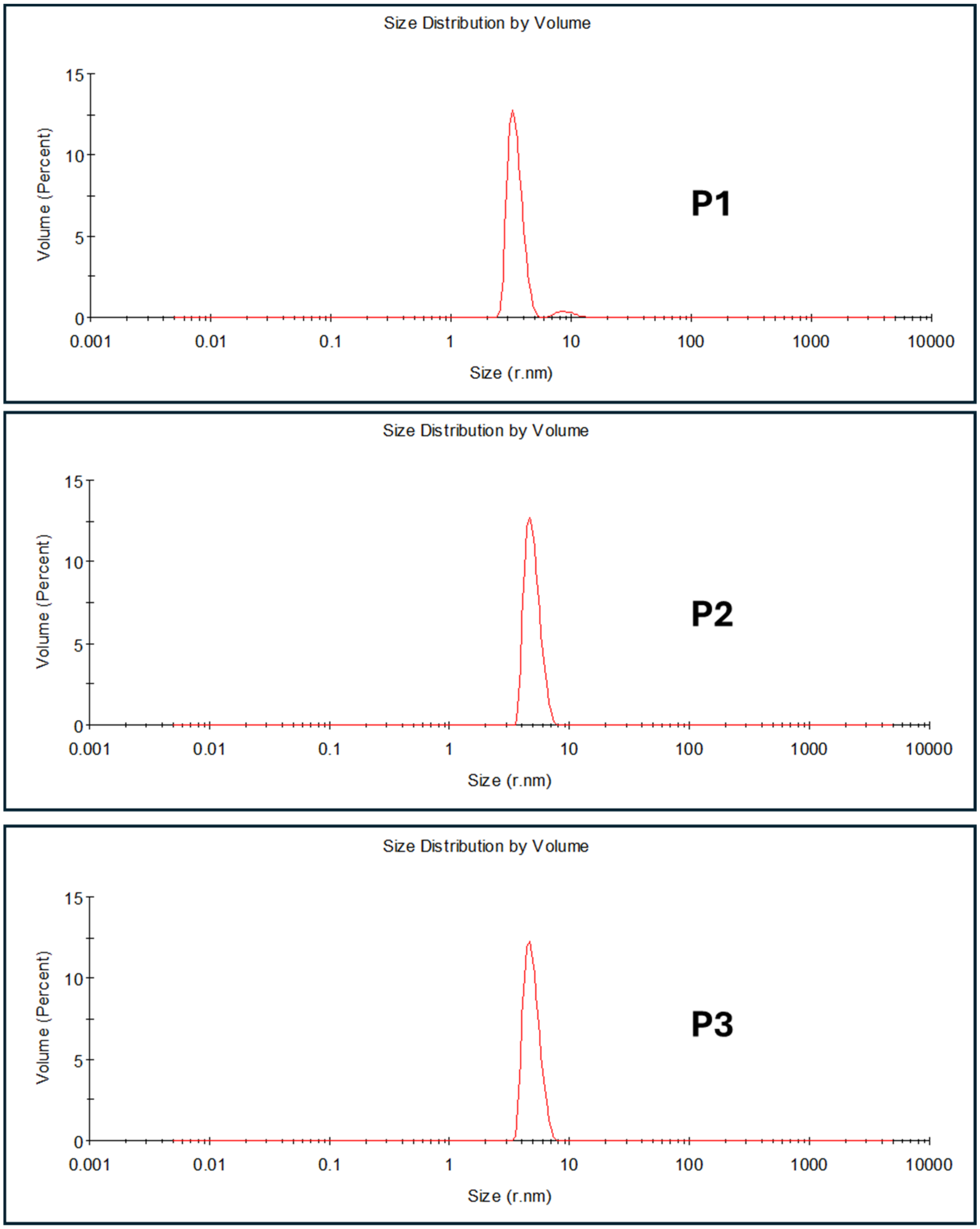

Figure S12: Dynamic light scattering measurements of **P1-P3**, [**P**] = 1 mg/mL in H_2O.

7. HPLC-UV

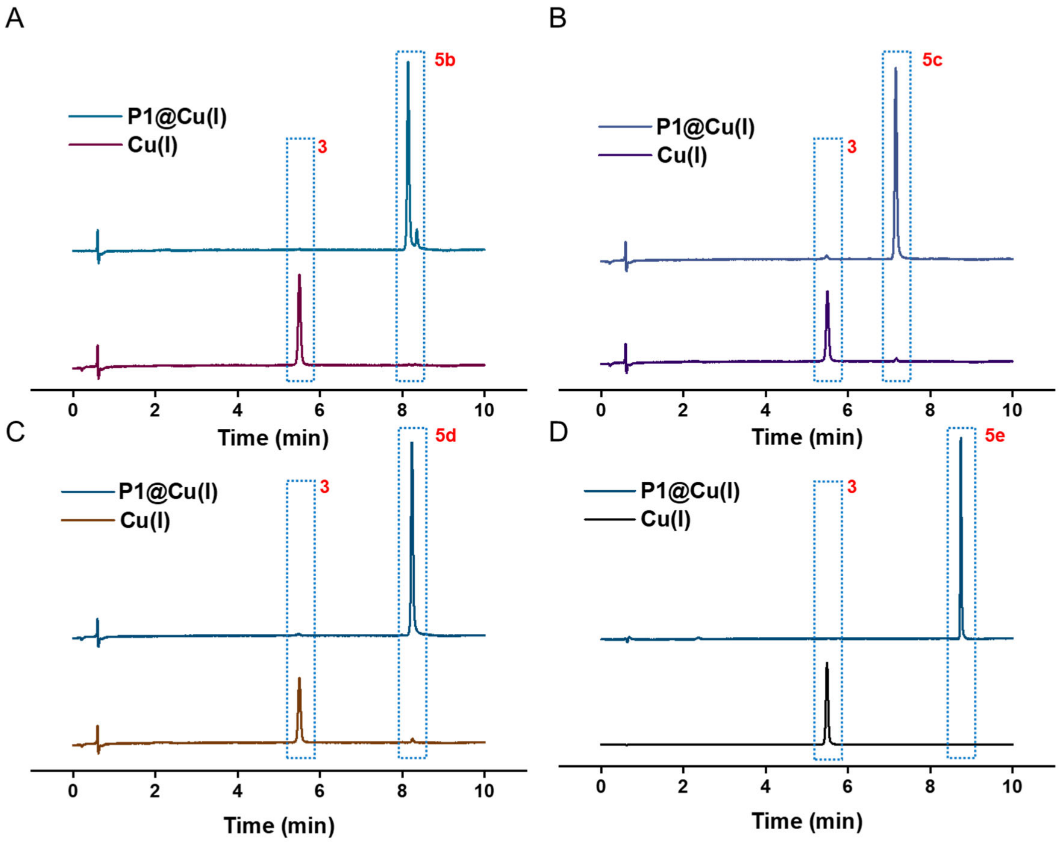

Figure S13: HPLC-UV chromatogram after CuAAC reaction between **3** and **4b-e** to form **5b-e** using **P1@Cu(I)** and **Cu(I)**, monitored after 10 min, at λ = 340 nm. Reaction conditions: in H_2O, [**Cu(I)**] = 10 μM, [**3**] = 100 μM, [**4b-e**] = 400 μM, [NaAsc] = 2 mM, [**P**] = ~600 nM, at room temperature.

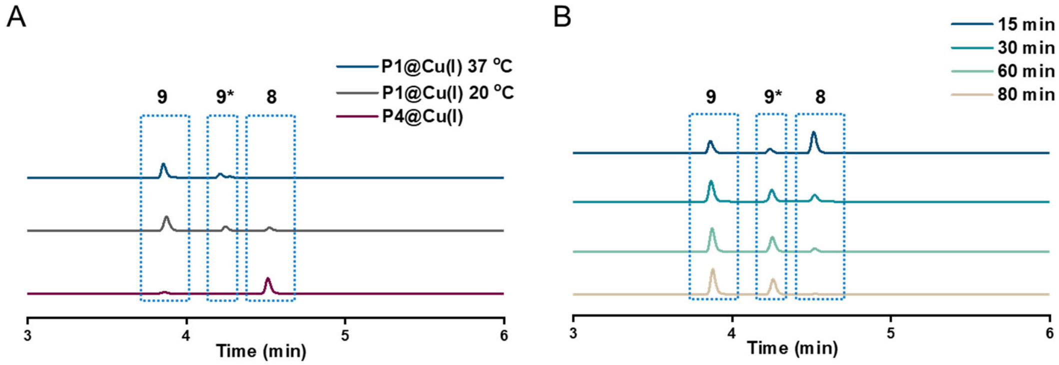

Figure S14: HPLC-UV chromatogram A) pro-rho **8** activation using **P1@Cu(I)**, *T* = 20 °C and 37 °C and **P4@Cu(I)**, *T* = 20 °C. Reaction conditions = in H_2O, [**Cu(I)**] = 2 μM, [**8**] = 30 μM, [NaAsc] = 2 mM, [**P**] = ~120 nM. B) pro-rho **8** activation using **P1@Cu(I)** over time, reaction

conditions = in H_2O, [**Cu(I)**] = 10 μM, [**8**] = 100 μM, [NaAsc] = 2 mM, [**P**] = ~600 nM. **9*** formed during pro-rho activation was also monitored using HPLC-MS, but was not detected as sharply as seen in UV, with $[M+H]^+$ = 510 and is expected to be the intermediate after depropargylation before the release of CO_2 stabilised by Na^+ from sodium ascorbate.

8. Ensemble kinetic studies

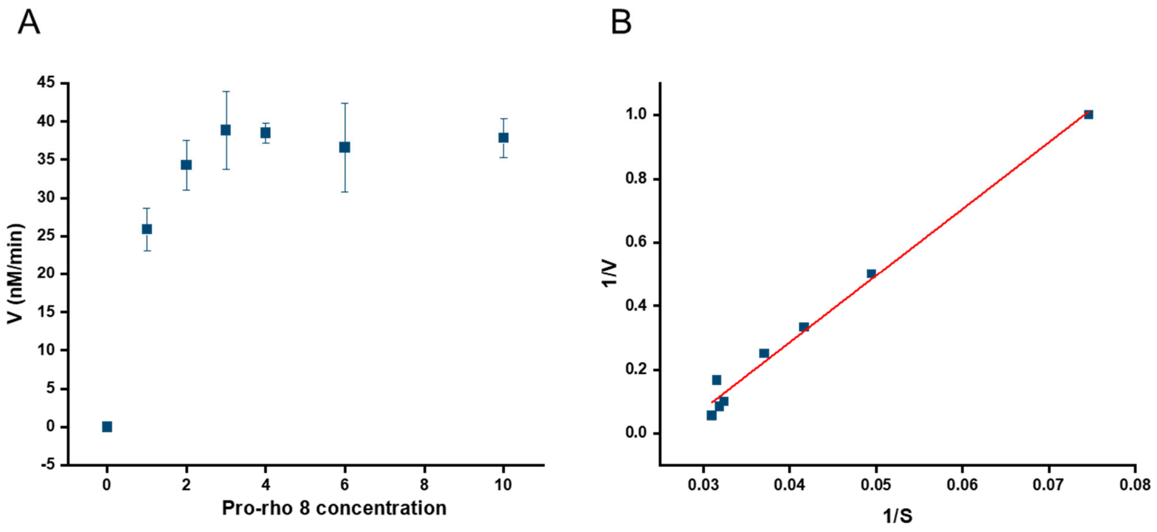

Figure S15: A) Change in the initial reaction rate of the depropargylation reaction of pro-rho **8** with the increase in substrate concentration using **P1@Cu(I)**, error calculated from three independent measurements. B) Lineweaver-Burk plot of reaction kinetics of the same reaction catalysed by **P3@Cu(I)**, rate monitored using fluorescence kinetic experiment (t_0 = 0 min and t_f = 10 min) and concentration of product determined from the calibration curve of rho **9**, [**P**] = 5 nM, in H_2O, T = 37 °C, pro-rho **8** concentration as indicated, Equation : $1/V = (K_m/V_{max} \times 1/[S]) + (1/V_{max})$, in the plot $y = 1/V$ and $x = 1/[S]$, $R^2 = 0.98$.

9. Single-particle kinetic studies

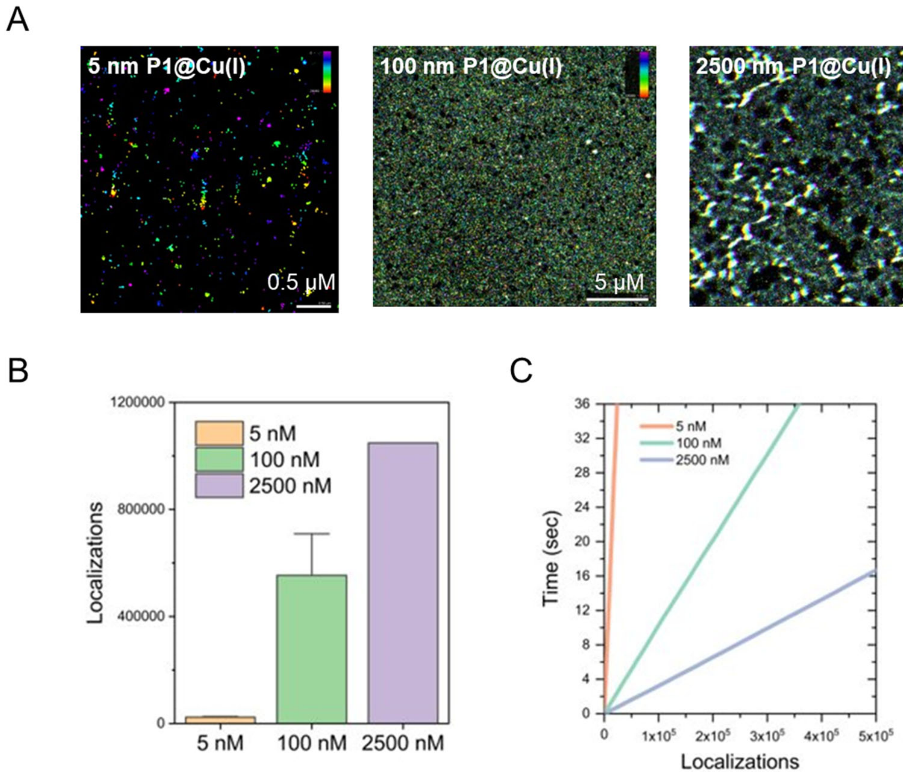

Figure S16: A) Time-sequence catalysis mapping using TIRF microscope monitoring the activation of pro-rho **3** by immobilised **P1@Cu(I)** in increasing concentrations B) Localisations of product molecules after catalysis measurements at different concentrations of **P1@Cu(I)** D) Localisation of product molecules overtime at different concentrations of **P1@Cu(I)**, [pro-rho **3**] = 1 μM, [NaAsc] = 1 mM, [**P**] = 5, 100 and 2500 nM.

10. Mechanism of Cu(I) catalyzed depropargylation reactions

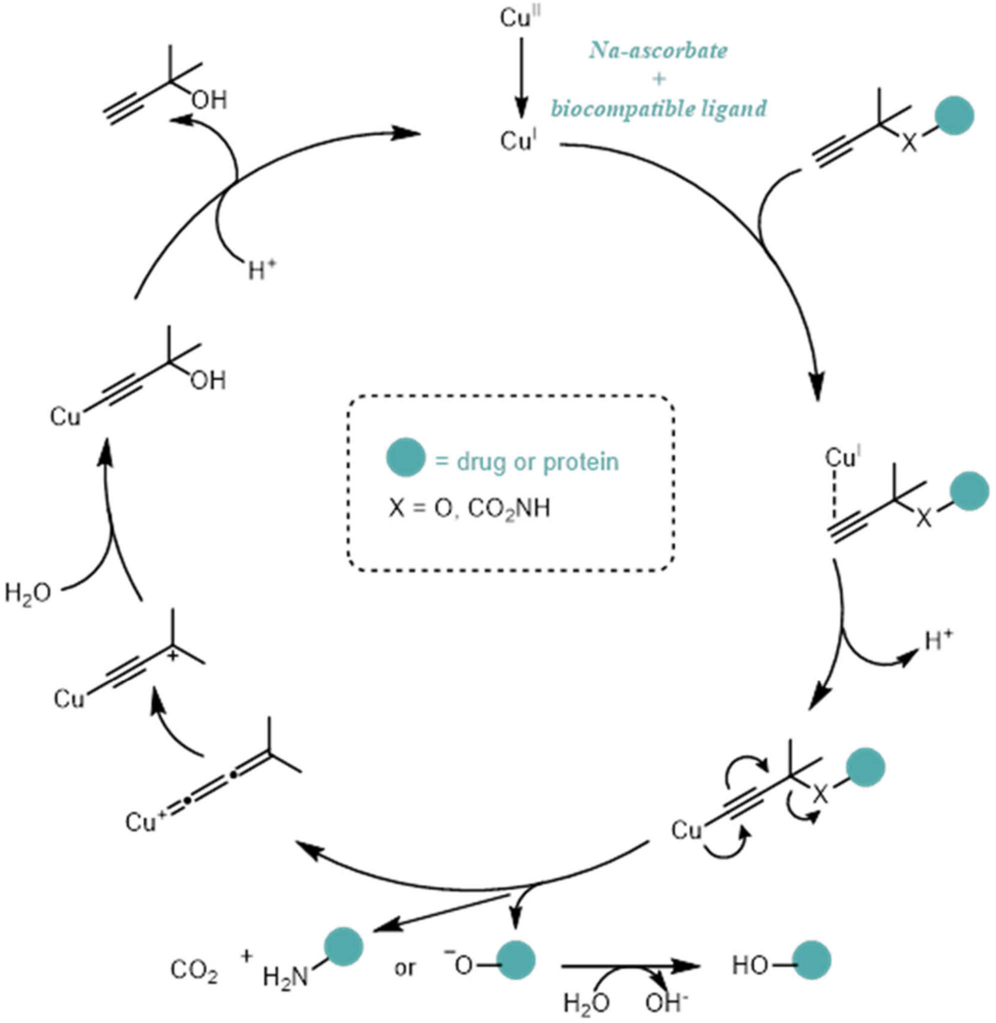

Figure S17: Reaction mechanism of Cu(I) catalyzed depropargylation reaction.[1]

11. References

1. Latocheski, E.; Dal Forno, G.M.; Ferreira, T.M.; Oliveira, B.L.; Domingos, J.B.; Bernardes, G.J.L.; et al. Mechanistic Insights into Transition Metal-Mediated Bioorthogonal Uncaging Reactions. *Chem Soc Rev* **2020**, *49*, doi:10.1039/D0CS00630K.
